# Efficacy of PACE4 pharmacotherapy in JHU-LNCaP-SM preclinical model of androgen independent prostate cancer

**DOI:** 10.1038/s41598-022-21593-7

**Published:** 2022-10-19

**Authors:** Nawel Mekdad, Thi Minh Hue Tran, Roxane Desjardins, Anna Kwiatkowska, Frédéric Couture, Robert Day

**Affiliations:** 1 Institut de Pharmacologie de Sherbrooke, Université de Sherbrooke, Sherbrooke, QC Canada; 2 Faculté de Médecine et des Sciences de la Santé, Université de Sherbrooke, Sherbrooke, QC Canada; 3 Département de Biochimie et Génomique Fonctionnelle, Université de Sherbrooke, Sherbrooke, QC Canada; 4 Faculté des Sciences, Département de Chimie, Université de Sherbrooke, Sherbrooke, QC Canada; 5 TransBIOTech, Lévis, QC Canada; 6 PhenoSwitch Bioscience, Sherbrooke, QC Canada

**Keywords:** Prostate, Target validation

## Abstract

Prostate cancer (PCa) is a complex disease progressing from in situ to invasive or metastatic tumors while also being capable of modulating its androgen dependence. Understanding how novel therapies are working across the different stages of the disease is critical for their proper positioning in the spectrum of PCa treatments. The targeting of proprotein convertase PACE4 (Paired basic Amino Acid-Cleaving Enzyme 4) has been proposed as a novel approach to treat PCa. Animal studies performed on LNCaP xenografts, an androgen-dependent model, already yielded positive results. In this study, we tested PACE4 inhibition on JHU-LNCaP-SM, a newly described androgen-independent model, in cell-based and xenograft assays. Like LNCaP, JHU-LNCaP-SM cells express PACE4 and its oncogenic isoform PACE4-altCT. Using isoform-specific siRNAs, downregulation of PACE4-altCT resulted in JHU-LNCaP-SM growth inhibition. Furthermore, JHU-LNCaP-SM responded to the PACE4 pharmacological inhibitor known as C23 in cell-based assays as well as in athymic nude mice xenografts. These data support the efficacy of PACE4 inhibitors against androgen independent PCa thereby demonstrating that PACE4 is a key target in PCa. The JHU-LNCaP-SM cell line represents a model featuring important aspects of androgen-independent PCa, but it also represents a very convenient model as opposed to LNCaP cells for in vivo studies, as it allows rapid screening due to its high implantation rate and growth characteristics as xenografts.

## Introduction

PCa is a worldwide health problem and is the second leading cause of cancer deaths in North American men. It is the type of cancer most often diagnosed in men^1^. Early-stage PCa depends on testosterone and dihydrotestosterone for growth and survival, which makes androgen ablation therapy efficient for tumor regression. Tumors that are not surgically removed and which are treated with anti-androgenic therapies eventually become androgen-independent, allowing them to overcome treatment and recur^2,3^. Chemotherapy is used with limited effects in androgen resistant PCa as tumors then develop resistance through clonal selection or neuroendocrine differentiation which further leads to different behavior and response to currently used therapeutic approaches^3^. In that sense, continued research on novel therapies remains relevant, especially in late-stages of the disease.

PCa research is also limited by the lack of a representative cellular model of the disease. Unless being derived into androgen independence after lengthy culture conditions or turned androgen-resistant by castrating animals after xenografts have developed, the LNCaP cell line is not the optimal model of advanced PCa^4^. Characterized by Horoszewicz et al., in 1983, the LNCaP cell line is a well-studied and often used model for in vitro and in vivo studies. The LNCaP cell line was first established from a lymph node isolated metastatic lesion of human prostate adenocarcinoma. It grows in vitro with a doubling time of about 60 h and can develop tumors, although with a poor take-rate (about 40–60%) at the injection site in athymic nude mice^5^. Thus, LNCaP cells are not ideal to model advanced PCa due to their androgen dependence and have disadvantages such as slow growth as semi-adherent cells which influences readout in various cell-based assays^5^. Other cell lines such as PC3 are widely used as an androgen independent model, but they mostly represent a neuroendocrine form of PCa^6^ and also lack PACE4 expression. Another example is the DU145 cells, which respond well to PACE4-inhibition^7^ but lack both androgen receptor and PSA expression and thus cannot be used as a metastatic model of androgen independent disease.

Working with a cell line having as much as possible biochemical characteristics specific to PCa and allowing studies to be carried out in a reasonable time frame represents an important asset for rapid drug screening. Furthermore, PCa heterogeneity in terms of aggressiveness, androgen sensitivity as well as tumor cell drivers hampers the development of a model mimicking the entire evolution of the disease^8^. In 2016, Castanares et al. identified a new LNCaP-derived cell line, namely JHU-LNCaP-SM^9^. This cell line was obtained by long-term serial passaging in a standard growth medium and differs from LNCaP by chromosome 23 pair. Instead of X, Y; this cell line was found to have X, X instead. This partial loss of the Y chromosome is common in immortalized cell lines and is associated with an increased risk of cancer in males and pathology aggressivity. Castanares et al., showed that this cell line is androgen-insensitive and readily forms subcutaneous tumors that can reliably metastasize to the lymph nodes in athymic nude mice. This cell line was proposed as a very powerful tool to study PCa and develop new treatments for advanced PCa.

PACE4 (gene name *PCSK6)* is a member of the proprotein convertase (PC) family of enzymes that have been shown to play a very pivotal role in PCa progression, firstly by its overexpression in PCa tissues^7,10^. Furthermore, PACE4 mRNA undergoes alternative splicing and polyadenylation resulting in two distinct isoforms, namely PACE4 (full length or PACE4-FL) and PACE4-alternative C-terminal (PACE4-altCT), the latter being an oncogenic form expressed by PCa cells^11^. Through its proteolytic activity, PACE4 mediates its role in PCa by the activation of protein precursors. In PCa cells, PACE4-altCT is routed differentially in the intracellular pathway where it remains and will cleave substrates that are not normally processed in normal prostate cells. Among the known PACE4 substrates, pro-Growth Differentiation Factor-15 (pro-GDF-15) has been identified as the first substrate of PACE4 in PCa^11^. GDF-15 belongs to the Tumor Growth-Factor β superfamily (TGF-β) and is known to mediate cell growth^12^. In PCa, increased concentrations of GDF-15 are associated with tumor progression. Due to the positive correlation between the level of GDF-15 and the advanced tumor stage, GDF-15 has been reported as a potential diagnostic and prognostic biomarker for PCa^13^. Other PACE4-substrates associated with PCa will likely be uncovered, but this is outside of the scope of the present manuscript.

A strong body of evidence positions PACE4 as a new target for drug development for PCa^14–16^. A PACE4-specific peptide inhibitor named C23 was developed and found to efficiently block PCa cells proliferation and progression^15^. Using siRNA silencing strategies specifically targeting individually each PACE4 isoform, the knockdown of PACE4-altCT but not the canonic isoform of PACE4 (PACE4-FL) results in inhibition of cell proliferation. Given the overexpression of this isoform in cancer cells and these findings, PACE4-altCT has been proposed as the target of C23 which mediate the compound pharmacological response in terms of cancer cell growth^11^. While there is cumulated evidence for PACE4-altCT as a therapeutic target in PCa, further efficacy studies using in vivo models of advanced PCa are needed to better position this new therapeutic avenue. These efforts have been hampered by PCa cell lines currently available. For advanced PCa, an androgen-independent cell line with high xenograft implantation rates would be ideal. An example of such a cell line is the DU145 cells, which well yield high tumor take-rate, are androgen-independent and express PACE4. However, when used in xenografts, they respond very poorly to *iv* drugs due to a barrier that forms around the implanted tumor, as well as poor vascularization and lack of metastatic potential.

The JHU-LNCaP-SM cell line shows most of the typical advanced PCa characteristics while being convenient in vivo. In the present study, we have performed a comparison between the LNCaP and the JHU-LNCaP-SM cell models in terms of PACE4 isoform expression, responsiveness to the PACE4 pharmacological inhibitor C23 and specific PACE4 isoform silencing in a cell-based proliferation and colony formation assay. The cell lines were also compared in terms of convenience (i.e., implantation rates/growth rates) when used as xenografts in athymic nude mice as well as in terms of pharmacological response to the C23 PACE4-inhibitor. In xenograft assay, tumor progression based on volumes and weights as well as using immunohistochemical markers for cell proliferation, quiescence and apoptosis such as, Ki67, P21, PARP and P27^KIP^ have been compared. Plasma analysis of PSA (prostate-specific antigen) as well as the PACE4-substrate GDF-15 were also compared. The results show that C23 and the specific PACE4-altCT siRNA both efficiently block cell proliferation in androgen-independent JHU-LNCaP-SM just like in LNCaP cells. Also, when used in vivo, C23 efficiently block JHU-LNCaP-SM xenograft progression as tumor. In comparison to LNCaP cells, the JHU-LNCaP-SM were much easier to culture and implant as xenografts. The JHU-LNCaP-SM cell line thus display all the required characteristics relevant to further PACE4 pharmacotherapy studies. Due to androgen independence we propose that the JHU-LNCaP-SM cell line could be a good model for advanced PCa and are certainly very useful for studies linked to PACE4 in PCa and especially for drug screening, in in-vitro studies as well as in animal models.

## Material and methods

### Cell culture

LNCaP cells were obtained from the American Type Tissue Collection (ATCC). JHU-LNCaP-SM cells were kindly provided by Dr Catherine A. Foss (University School of Medicine, Baltimore, Maryland). LNCaP and JHU-LNCaP-SM cells were both grown in RPMI-1640 media (Wisent Bioproducts, St Bruno, QC) supplemented with 10% fetal bovine serum (FBS; Wisent Bioproducts). Mycoplasma testing was done after every new cryotube thawing.

### Reverse transcription and quantitative polymerase chain reactions

Total RNA was extracted from cells using the Qiagen RNA Isolation MiniKit (Qiagen, Valentia, CA). For reverse transcription, 1 µg of RNA was DNase I–treated (Invitrogen), reverse-transcribed using Superscript II reverse transcriptase kit (Invitrogen), and RNase H-treated before performing SYBR green quantitative PCR (qPCR). The reaction mixtures combined cDNA with a master mix containing primers. The PCR profile was one cycle for 10 min at 95 °C, 40 cycles of 30 s at 95 °C, 1 min at 60 °C, and 30 s at 72 °C. All primers used are listed in Supplementary Table 1. All reactions were performed in duplicate. For all PCRs, standard curves, dissociation curves, and migration of PCR products on acrylamide gels were done to confirm the specificity of the products. Relative expression levels were calculated using β-actin as the housekeeping gene with the formula (1 + amplification efficiency)^−CT^.

### C23 synthesis

C23 peptide was synthesized manually by a combination of solid-phase peptide synthesis and solution synthesis, as detailed in Kwiatkowska A. et al.^15^. All peptides were purified by reverse-phase HPLC and compound identification and purity were assessed by analytical HPLC. High-resolution mass spectrometry was used to confirm the identity of the pure products.

### MTT proliferation assay

The cell proliferation was measured by the colorimetric MTT assay (thiazolyl blue tetrazolium bromide; Sigma-Aldrich, Oakville, Ontario, Canada). Cells were seeded in 96-well plate (BD Biosciences, Mississauga, Ontario, Canada) with 100 µl of a 3.5 × 10^4^ cells/mL cell suspension in complete growth medium (10% FBS) for LNCaP cells or 1.0 × 10^4^ cells/ml cell suspension in complete growth medium (10% FBS) for JHU-LNCaP-SM cells. The following day, cells were treated with C23 in triplicate for each concentration (1.5 mM, 1 mM, 0.75 mM, 0.5 mM, 0.25 mM, 0.1 mM, 0.01 mM diluted in sterile water 1% DMSO). 90 µL of fresh complete growth medium (10% FBS) is added and 10 µL of C23 peptide is added in each well to yield the final indicated concentrations. The plates were incubated at 37 °C, 5% CO_2_ for 72 h before 25 µL of an MTT solution (5 mg/ml in PBS 1 ×) was added to each well for another 5 h at 37 °C/5% CO_2_. The medium was then carefully discarded, and the cells were solubilized with 100 µl of DMSO. The absorbance was measured at a wavelength of 560 nm in a microplate reader (SpectraMax190; Molecular Devices, Sunnyvale, CA).

To evaluate response to androgen, cells were seeded in 6 mirror 96-well plate (BD Biosciences, Mississauga, Ontario, Canada) with 100 µl of a 1.75 × 10^4^ cells/mL cell suspension in complete growth medium (10% FBS) for LNCaP cells or 5.0 × 10^4^ cells/mL cell suspension in complete growth medium (10% FBS) for JHU-LNCaP-SM cells. The following day, cells were treated with DHT (Sigma Aldrich) in triplicate for each concentration (10^–6^ M, 10^–7^ M, 10^–8^ M, 10^–9^ M, 0 M). 90 µL of fresh complete growth medium (10% FBS) is added and 10 µL of DHT (diluted in complete medium) is added to each well. The plate is incubated at 37 °C, 5% CO_2_. Each day, for 6 days, one plate was revealed by MTT as described above.

### Colony formation assay

Cells were plated at low density in a 6-well plate (750 cells/well for LNCaP and 100 cells/well for JHU-LNCaP-SM) and maintained in complete medium at 37 °C, 5% CO_2_. Every 3 days, complete medium was discarded and refresh and replenished and cells were treated with C23 at 50 µM diluted in complete medium and allowed to form colonies for 10–14 days. Colonies were fixed and stained with 10 mg/mL crystal violet/10% formaldehyde solution for 30 min. Excess of staining solution was removed carefully with distilled water and the plates were dried overnight before scanning with Li-Cor Odyssey Infrared Imaging System (Li-Cor Biosciences, Lincoln, NE). Scanned images were analyzed with ImageJ software 1.37v to measure the total particle area.

### RNA silencing

Cells were plated in a 6-well plate (500,000 cells/well for LNCaP and 150,000 cells/well for JHU-LNCaP-SM). Cells were maintained in complete medium at 37 °C, 5% CO_2_. After 24 h, cells are transfected with siRNA specifically targeting PACE4-FL or PACE4-altCT (respectively GACAAATATTTCATATATT[dT][dT] & 5′GGAAGGAUGUACUCACAUC[dT] [dT] purchased from Sigma Aldrich). Transfections were performed using 0, 5, 10 and 25 nM in Opti-MEM, reduced serum medium (Gibco) and lipofectamine RNAiMax transfection reagent (Invitrogen) for 6 h before adding medium with FBS. After 24 h, transfection media was removed and 2 mL of complete medium is added. Following an additional 24 h, transfected cells were collected for plating in another 6-well plate as described above for colony formation assay.

### Xenograft assays

All studies were performed in accordance with the Canadian Council on Animal Care guidelines and were approved by the University of Sherbrooke animal Ethical Care Committee in accordance with ARRIVE guidelines. For tumor xenografts, trypsin-harvested JHU-LNCaP-SM or LNCaP cell suspension was mixed with an equal volume of Matrigel (BD Biosciences, Bedford, MA) on ice and subcutaneously injected (100 μL) at 2 different sites (left and right shoulders) in athymic nude (Nu/Nu) male mice (Charles River Laboratories, St-Constant, Canada) at a density of 2 × 10^6^ cells/site. Mice were housed under pathogen-free conditions, in an environment maintained at 21 ± 0.5 °C with controlled humidity and a light–dark cycle. Autoclaved food and water were provided ad libitum. Manipulations were performed in a biosafety cabinet. Tumors were measured and the tumor volumes were calculated from the equation: $Lenght \times {width}^{2} \times \frac{\pi }{6}$. When tumors were manually palpable and reached 50–100 mm^3^, mice were divided in two groups. Tumors measurements were realized under isoflurane using a micrometric caliper. Mice were then injected intraperitoneally with C23 (2 mg/kg) from a stock solution at 0.2 mg/mL or with vehicle (2% DMSO NaCl 0.8%) each day for 24 days. Blood sampling (50–75 μL) from the saphenous vein was performed weekly and plasma was stored at − 80 °C for Prostate-Specific-Antigen (PSA) levels determination using a PSA ELISA assay (ClinPro International, USA). At the end of the experiment, on day 24 (JHU-LNCaP-SM xenograft) or day 30 (LNCaP xenograft), plasma was prepared from blood collected by cardiac exsanguination under ketamine: xylazine (0.2 mL/100 g IP dosing of 87:13 mg/kg respectively) to further evaluate GDF-15 level by ELISA assay (R&D Systems, USA). Plasma was stored at − 80 °C until analysis.

### Immunohistochemistry analysis

Upon mice sacrifice, tumors were collected and fixed in paraformaldehyde solution (4%) and paraffin-embedded (FFPE). After two baths with ethanol 70%, tumors were paraffin embedded and slides with tumor tissue were realized. Slices were deparaffinized by successive incubation for 5 min in the following solutions xylene (twice), ethanol 100% (twice), 95%, 85%, 70%, 50%, and 30%, UltraPure Water (twice), and 10 mM citrate buffer pH 6.0. Antigen retrieval was performed autoclaving in 10 mM citrate buffer pH 6.0 for 45 min (16 psi, 250°F). After cooling at room temperature, immunostainings were performed using the Peroxidase Detection Kit (Pierce). Tissue sections were incubated overnight at 4 °C with the primary antibodies diluted in BSA 5% in TBST. The primary antibodies used and their specific conditions are listed in Supplementary Table 2. Following washes, slides were incubated with a secondary HRP-conjugated antibody (anti-rabbit HRP from Bio-Rad diluted accordingly in TBST with BSA 5%, 1/500). Slides were then counterstained with Harris hematoxylin (Sigma-Aldrich) and further scanned with a Nanozoomer (Hamamatsu, Hertfordshire, UK) using Nanozoomer Digital Pathology software. Several fields (3 per slide) were manually counted with QuPath Software for positive and negative cells to calculate a positivity index (positive/total cells).

### Western Blot analysis

For cell lysates, LNCaP and JHU-LNCaP-SM cells were seeded in 100 mm plates (JHU-LNCaP-SM: 1 × 10^6^ cells/dish; LNCaP: 1.5 × 10^6^ cells/dish). After 48 h of incubation at 37 °C in 5% CO_2,_ total proteins were extracted with radio-immunoprecipitation assay buffer (RIPA buffer) supplemented with protease inhibitors (Complete Mini; Roche). The samples were incubated for 30 min on ice and subsequently centrifuged for 30 min at 13.000 rpm at 4 °C. The sample protein concentrations were determined using the Bicinchoninic Acid Protein assay kit (Pierce, Rockford, IL) and 40 μg of protein samples were separated on a sodium dodecyl sulfate–polyacrylamide gel electrophoresis 10% (SDS-PAGE).

To produce conditioned media, cells were seeded in 100 mm plates (JHU-LNCaP-SM: 2.0 × 10^6^ cells/dish; LNCaP: 2.5 × 10^6^ cells/dish in complete growth media) and incubated for 24 h at 37 °C in 5% CO_2_. The next day, the growth media were replaced with 7 mL of serum-free RPMI medium and incubated at 37 °C in 5% CO_2_ for 48 h. Conditioned media was collected and centrifuged for 10 min at 3000 rpm at 4 °C. Aliquots were flash-frozen in liquid nitrogen, lyophilized for 48 h and reconstituted in 100 µL of urea 8 M: loading buffer (1:1). 30 µL were loaded on a SDS-PAGE 15%. Coomassie blue staining was routinely performed to control for protein loading. Each SDS-PAGE is transferred to a nitrocellulose membrane (Hybond; GE Healthcare, Chalfont St Giles, United Kingdom). Before immunodetection, membranes were blocked with 5% BSA in a 0.1% Tween-TBS solution. Membranes were then incubated with anti-actin (NeoMarkers, Fremont, CA), anti-GDF15 primary antibody overnight at 4 °C followed by incubation with an anti-rabbit or anti-mouse IgGs coupled to IRDye800 (LI-COR Biosciences, Lincoln, NE). Immunodetection was then performed using an infrared imager (Odyssey Imager, LI-COR Biosciences). Beta-actin was used as a loading control.

### Statistical analysis

All experiments were repeated at least three times, and the results were expressed as mean ± SEM. Statistical analyzes were performed using GraphPad Prism version 9.0. Student t-tests were used to calculate P-values.

## Results

### JHU-LNCaP-SM cells express PCs and PACE4 isoforms

**Figure 1:**
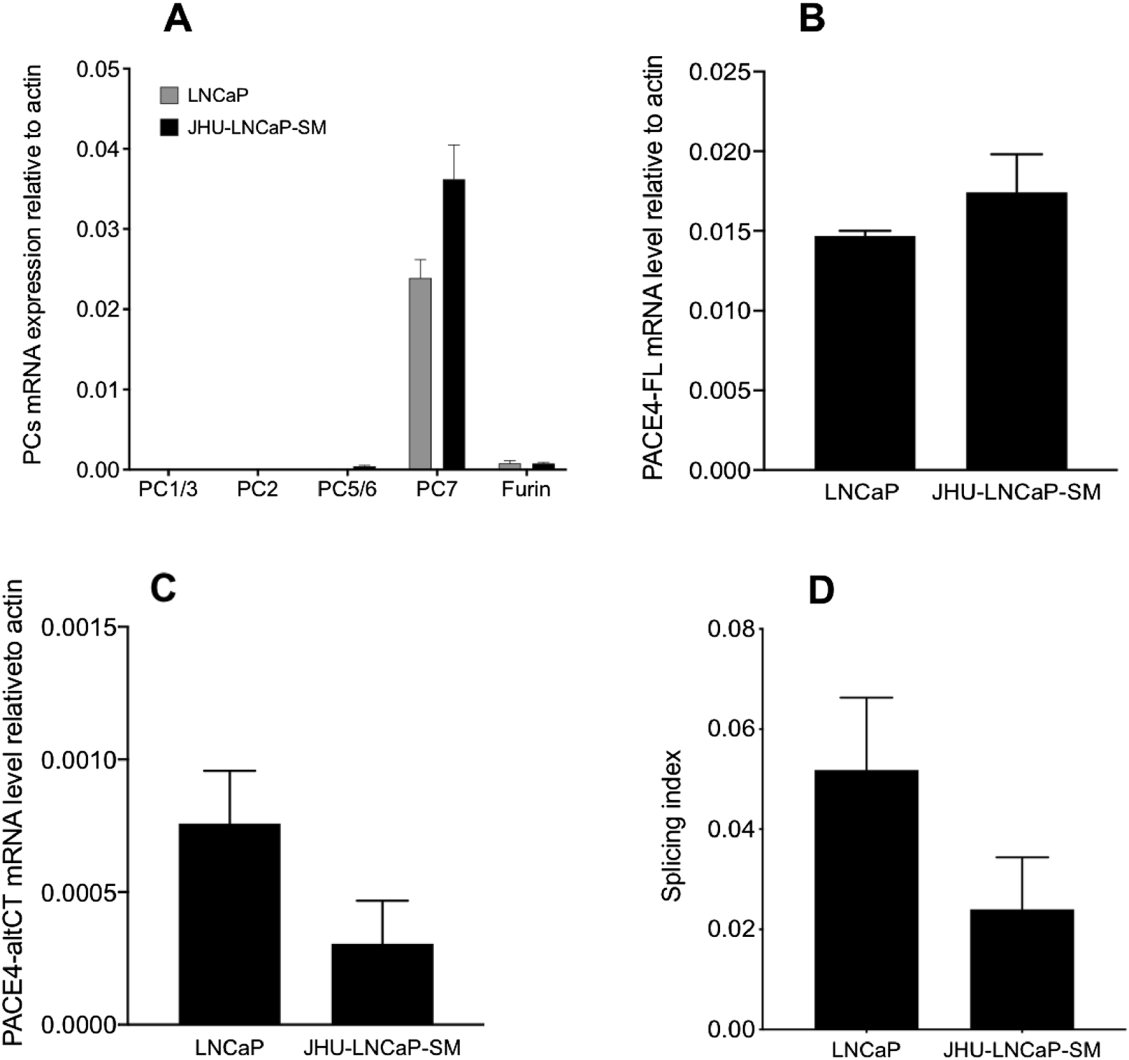
PACE4 splice variants and other PCs expression in JHU-LNCaP-SM. (**A**) Relative mRNA expression levels of PCs in LNCaP and JHU-LNCaP-SM cell lines. (**B**) PACE4-FL and (**C**) PACE4-altCT mRNA expression levels in LNCaP and JHU-LNCaP-SM cell lines (**D**) Splicing index (ratio of PACE4-altCT/FL) determined by RT-qPCR on the LNCaP and JHU-LNCaP-SM cells. Data are means ± SEM.

The overexpression of PACE4 in PCa has been documented, and a PACE4 mRNA terminal exon splicing event yield the expression of PACE4-altCT isoform in cancer cells^11^. The expression of PACE4 isoforms in JHU-LNCaP-SM was first assessed to ensure its expression in relation to all other members of the proprotein convertase family. As a control, we included LNCaP cells, although these have already been extensively studied for PC expression previously^10^. Similarly, to LNCaP, JHU-LNCaP-SM expresses furin and PC7 at rather similar levels (Fig. 1A) in contrast to PC5/6, which is non-detectable in LNCaP cells. Importantly, both PACE4-FL (Fig. 1B) and PACE4-altCT (Fig. 1C) mRNA are found in JHU-LNCaP-SM and LNCaP cells. Finally, PACE4-FL expression levels are higher than PACE4-altCT levels in both JHU-LNCaP-SM and LNCaP cell lines (Fig. 1D).

### JHU-LNCaP-SM cell line is androgen-independent and sensitive to both pharmacological and mRNA silencing of PACE4-altCT

**Figure 2:**
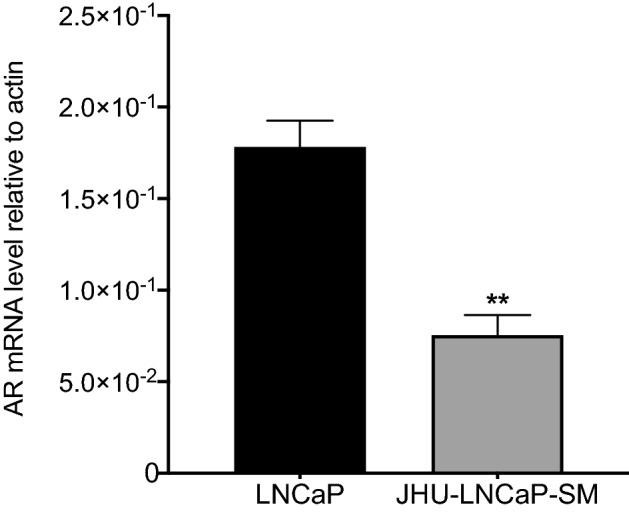
RT-qPCR measuring androgen receptor (AR). Relative mRNA expression of AR in LNCaP and JHU-LNCaP-SM cell line using actin as a normalizer housekeeping gene. Data are means ± SEM (n = 4). **p < 0.01 from a Student t-test.

An important feature of the JHU-LNCaP-SM cell line is its androgen-independent growth. JHU-LNCaP-SM and LNCaP cells were treated with increasing concentrations of dihydrotestosterone (DHT) and evaluated in terms of growth using an MTT assay over 6 days (see Supplementary Fig. 1). The LNCaP cells showed dose-dependent proliferation whereas JHU-LNCaP-SM cells were unaffected, thus confirming their androgen independence. As previously reported, this independence is present despite expression of androgen receptor (AR; Fig. 2), although expressed about threefold less in terms of mRNA than in LNCaP^9^.

**Figure 3:**
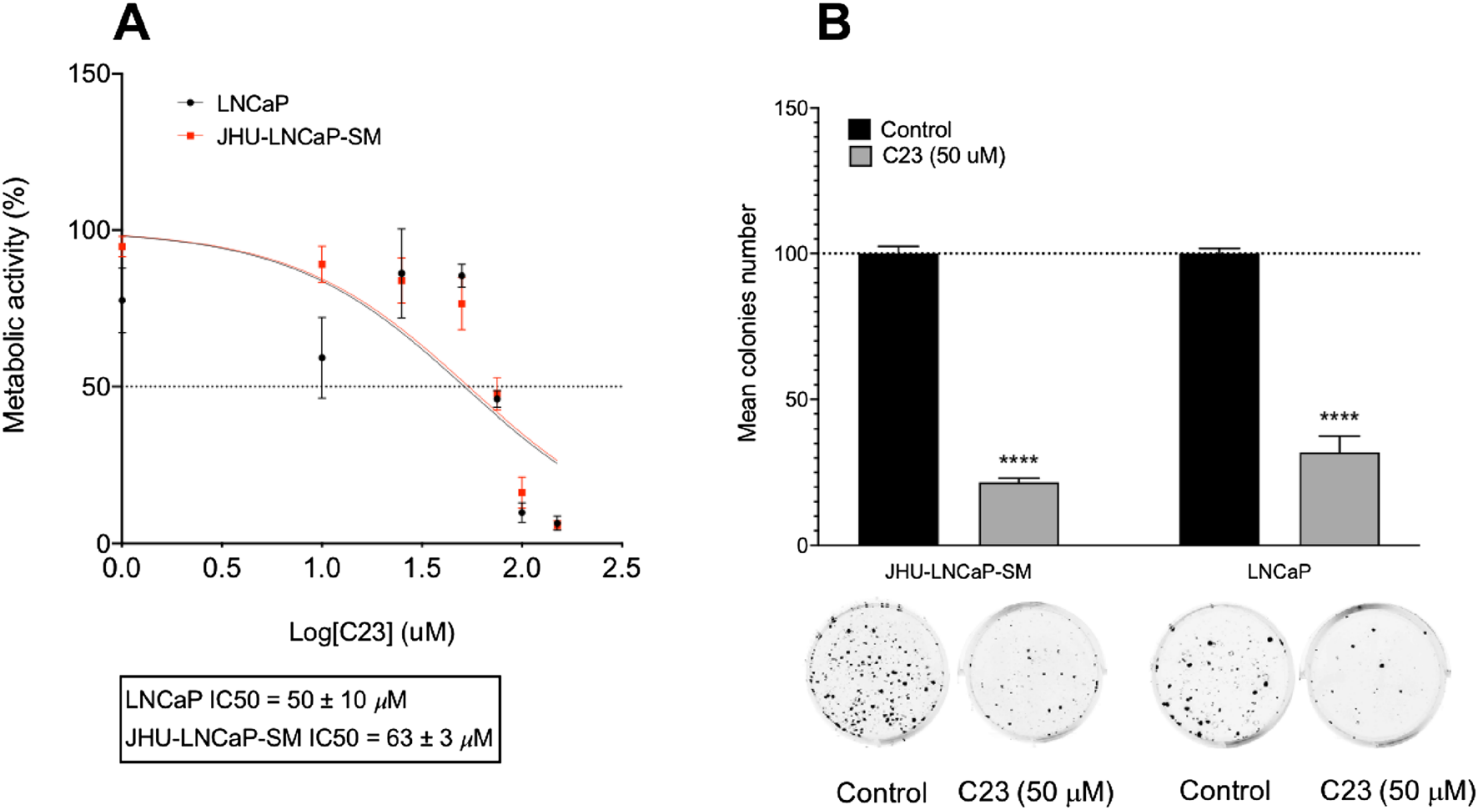
C23 PACE4 peptide inhibitor blocks JHU-LNCaP-SM cells proliferation and clonogenic potential. (**A**) Dose–response MTT proliferation assays in LNCaP and JHU-LNCaP-SM with IC_50_ values. (**B**) Colony formation assays on LNCaP and JHU-LNCaP-SM cell lines after C23 treatment (50 mM) with representative images of colonies after 14 days. Data are means ± SEM (n = 3–6). ****p < 0.0001 from a Student t-test.

PACE4 inhibition is known to induce cell growth inhibition and hinder clonogenic potential in PCa cells^11,16^. To establish the degree of response to PACE4 inhibitor of these cells, C23 compound was assayed in MTT proliferation assays. C23 treatment resulted in antiproliferative effects on both LNCaP and JHU-LNCaP-SM PCa cells, with IC_50s_ of 50 ± 10 μM and 63 ± 3 μM respectively (Fig. 3A). Based on these IC_50s_ values, the 50 µM dose was used to perform clonogenic assays^15^. C23 at 50 µM, also strongly reduced the clonogenic potential both cell types (Fig. 3B) with 79% and 69% less colony formation in JHU-LNCaP-SM and LNCaP cells, respectively.

**Figure 4:**
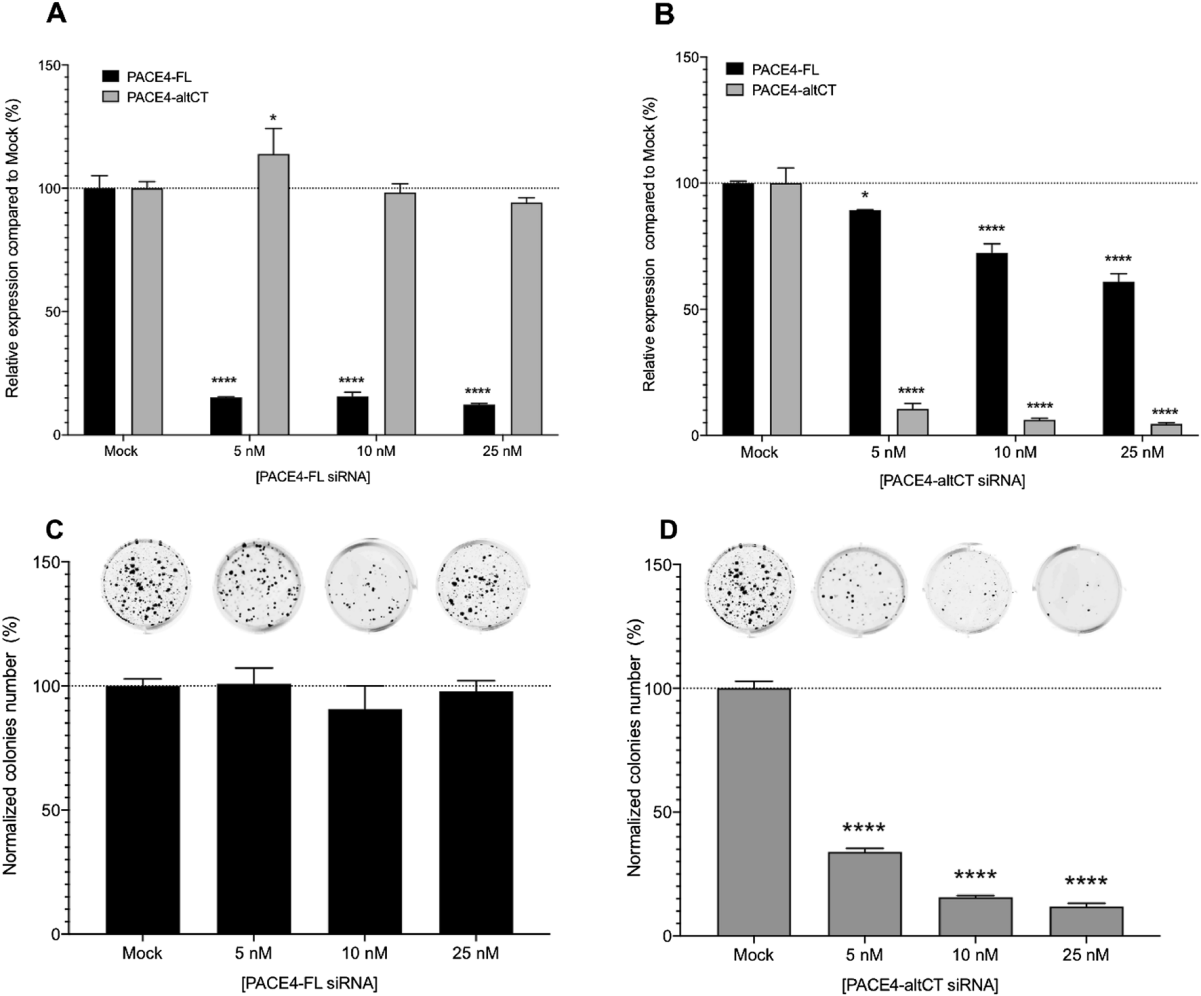
PACE4-altCT splice variant silencing results in cell growth inhibition in JHU-LNCaP-SM cells. (**A**) PACE4 isoforms silencing determined by RT-qPCR with different concentrations of specific siRNA targeting PACE4-FL and (**B**) PACE4-altCT in JHU-LNCaP-SM cells using actin as housekeeping gene (n = 2 in duplicate). (**C**) Colony formation assay with representative wells of JHU-LNCaP-SM silenced for PACE4-FL and (**D**) PACE4-altCT at 5, 10 or 25 nM. Data are means ± SEM (n = 3). ****p < 0.0001 from a Student’s t-test.

C23 is a potent inhibitor of both PACE4 isoforms^15^. It was previously reported that PACE4-altCT, but not PACE-FL, is responsible for the proliferation and growth of cancer cells. Thus, the specific role of each isoform was investigated using siRNA specific to each splice variant to knockdown each isoform separately. Following transfection and mRNA transcript analysis by RT-qPCR, PACE4-FL siRNA induced a dose-dependent decrease in the relative expression of its mRNA (Fig. 4A) with a reduction of 85% or more at 5 nM with no effect on the other splice variant levels. Similar silencing levels are obtained using siRNAs specific to PACE4-altCT which yield 88% gene silencing for this isoform with 5 nM and up to 96% with 25 nM, although it slightly reduced PACE4-FL levels (maximal reduction of 42% observed using 25 nM siRNA; Fig. 4B).

In the clonogenic assay, silencing of PACE4-FL did not prevent cells to form colonies at each dose (5, 10, 25 nM) (Fig. 4C) whereas PACE4-altCT silencing inhibited in a dose-dependent fashion colony formation resulting in more than 85% reduction of colony numbers with 25 nM PACE4-altCT siRNA (Fig. 4D). These results are consistent with the results obtained in the LNCaP parental cells^11^.

### GDF-15 expression in JHU-LNCaP-SM is reduced compared to LNCaP cells

**Figure 5:**
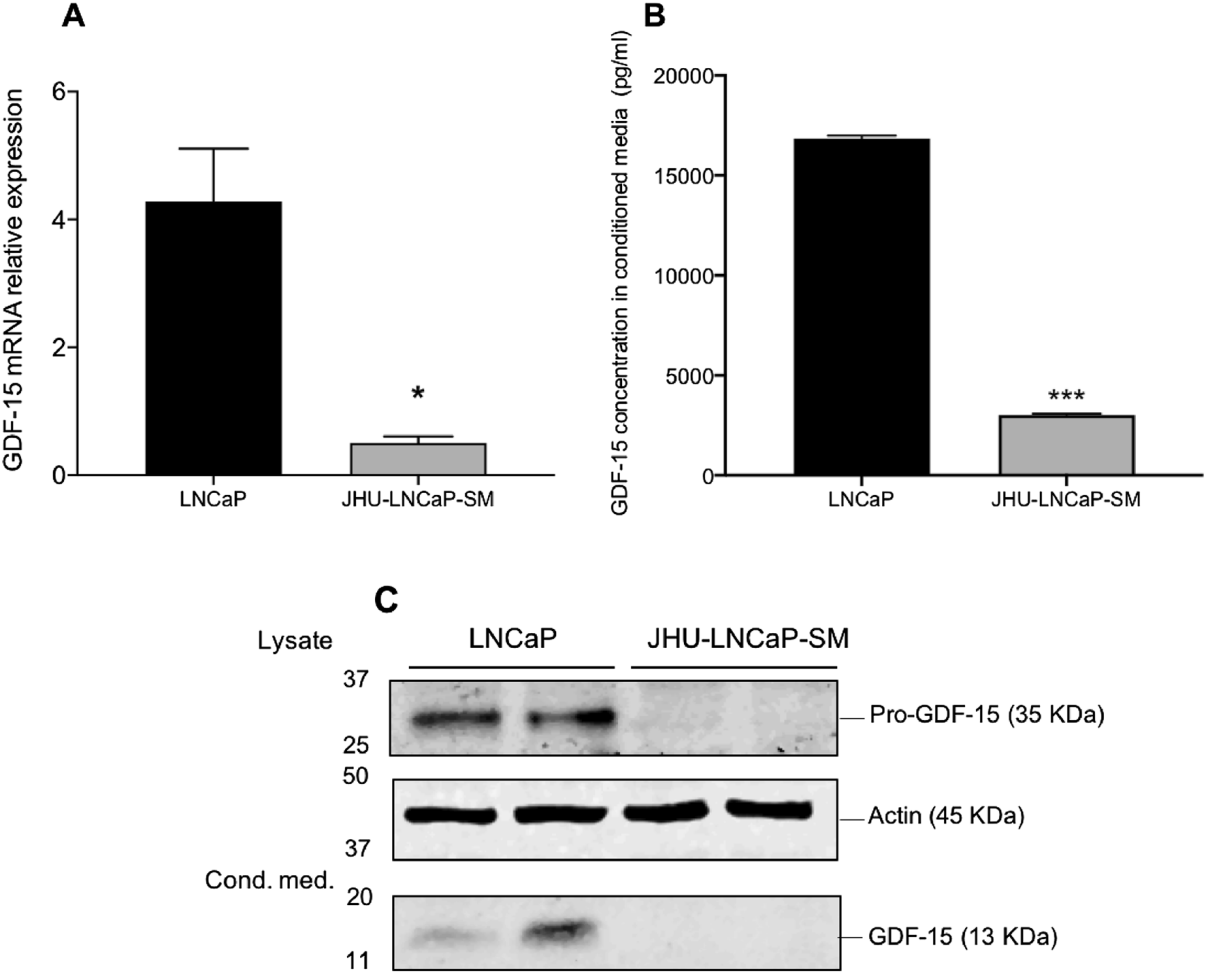
Expression of pro-GDF15 and GDF15 in LNCaP and JHU-LNCaP-SM cell lines. (**A**) RT-qPCR analysis of GDF-15 mRNA in LNCaP and JHU-LNCaP-SM cells using actin as a housekeeping gene, (**B**) GDF-15 concentration in conditioned media determined by ELISA assay **(C)** GDF15 and pro-GDF-15 levels in conditioned media and cell lysates respectively (two different gels). Two biological replicates are loaded side-by-side for each cell line. Data are means ± SEM. *p < 0.05 and ***p < 0.001 from a Student’s t-test.

GDF-15 has been identified as a PACE4-altCT substrate in LNCaP cells that mediates, at least in part, PACE4-altCT activity in relation to PCa cell growth and proliferation^11^. To compare LNCaP with JHU-LNCaP-SM cells, GDF-15 mRNA expression analysis was performed by RT-qPCR. GDF-15 mRNA levels were found to be significantly lower (about 10 fold) than in LNCaP where levels are very high (Fig. 5A).

GDF-15, which is expressed as a 35 kDa precursor (pro-GDF-15) that is further processed and secreted to a 26 kDa form (homodimer migrating at 13 kDa under denaturing conditions). GDF-15 concentrations were determined in the conditioned media by ELISA assays of both cell lines and showed a pattern similar to the one predicted from mRNA analyses (3076 compared to 16,700 pg/mL for JHU-LNCaP-SM and LNCaP respectively) (Fig. 5B). When analyzed at protein levels by Western Blot of both cell lysate and conditioned medium to visualize pro-form and mature forms of GDF-15, the difference between both cell lines was clearly demonstrated (Fig. 5C).

### C23 PACE4 inhibitor is effective at blocking JHU-LNCaP-SM and LNCaP xenograft growth in athymic nude mice but displays a distinct response on circulating PSA and GDF-15

**Figure 6:**
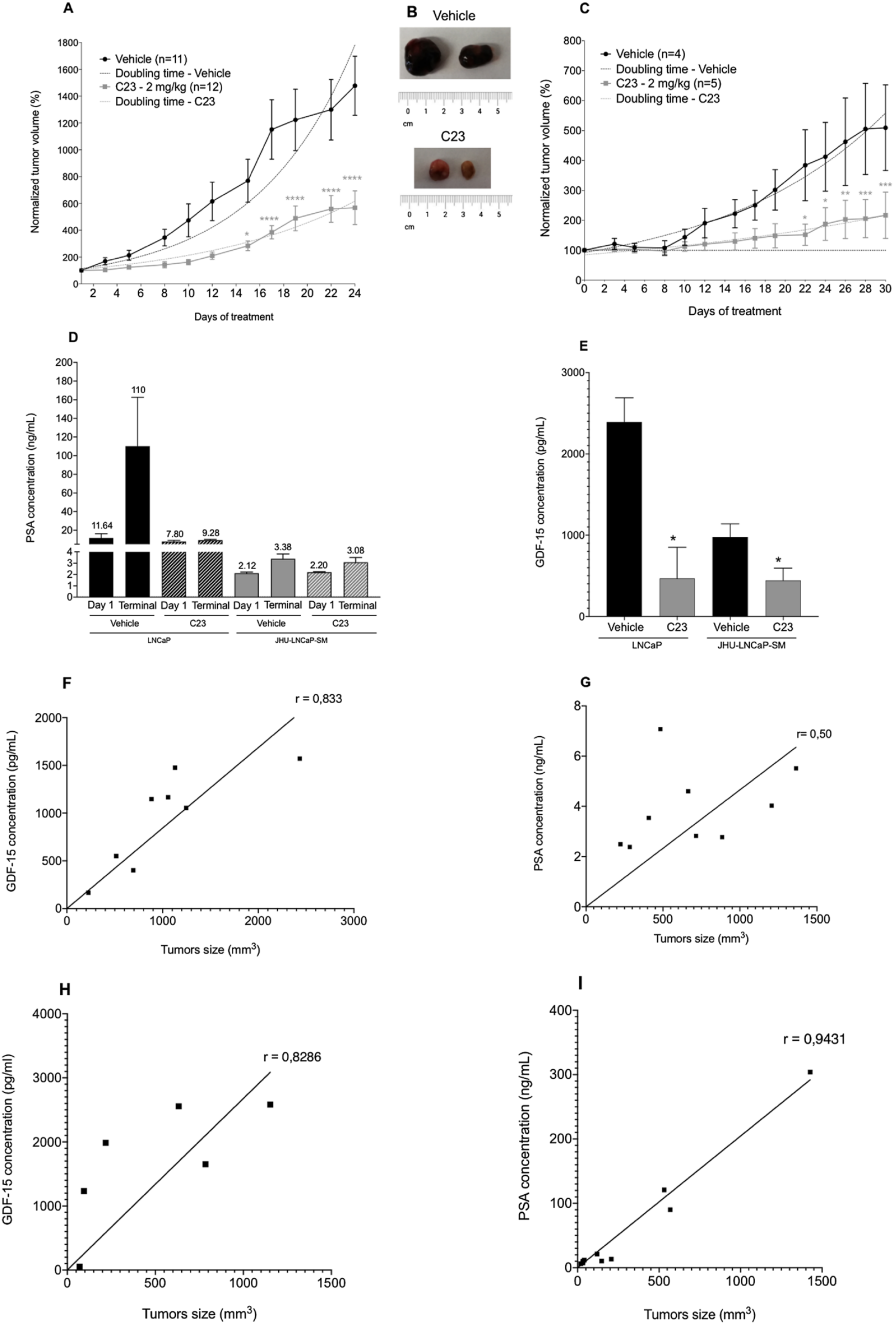
JHU-LNCaP-SM and LNCaP xenograft response to C23 administration. (**A**) JHU-LNCaP-SM relative xenograft size evolution over the course of treatment, the fits in transparency over the data show the equation used for doubling-time calculation. (**B**) Hemorrhagic appearance of JHU-LNCaP-SM tumors after 24 days of treatment with vehicle solution (IP) or C23 (2 mg/kg, IP). (**C**) LNCaP relative xenograft size evolution over the course of treatment, the fits in transparency over the data show the equation used for doubling-time calculation (**D**) PSA plasma concentrations at the start (Day 1) and the end (terminally sampled) of xenograft experiments (JHU-LNCaP-SM; 24 days; n = 6/group), LNCaP; 30 days; n = 5/group). The mean concentrations are indicated above each bar on the histogram in ng/mL (**E**) GDF-15 concentrations in mice plasma at the end of xenograft LNCaP assay (vehicle group n = 3; C23 group n = 3) and JHU-LNCaP-SM assay (vehicle group n = 9; C23 group n = 6) experiment evaluated by ELISA assay. (**F**) Spearman correlation between plasma GDF-15 (n = 8) or (**G**) PSA (n = 11) concentrations and xenograft tumors sizes from JHU-LNCaP-SM cell line. (**H**) Spearman correlation between plasma GDF-15 (n = 6) or (**I**) PSA (n = 9) concentrations and xenograft tumors sizes from LNCaP-SM cell line. Data are means ± SEM. *Indicates p-value < 0.05, **< 0.01, ***< 0.0001, ****< 0.0001 from a Student t-test.

To establish the sensitivity of JHU-LNCaP-SM xenografts to the PACE4 peptide inhibitor, the cells were inoculated subcutaneously in mice and were daily treated for 24 days with vehicle or with the C23 peptide (2 mg/kg) by intraperitoneal injection upon tumor formation. The take rate is variable depending on the cell line.The JHU-LNCaP-SM cell line forms tumors more easily with a take rate of 86% and 37% for LNCaP cell line. Intraperitoneal injection of C23 peptide significantly inhibited tumor progression from the 15th day of treatment until the end of the study (24 days) compared with the vehicle group (C23-doubling time: 9.1 days vs vehicle-doubling time: 5.8 days; Fig. 6A). At the end of the experiment, JHU-LNCaP-SM xenografts treated with C23 were 38% smaller than vehicle-treated ones and were visually less hemorrhagic (Fig. 6B). LNCaP xenografts were treated the same over a 30-days period (Fig. 6C). Compared with the vehicle group, LNCaP xenograft displayed a doubling time of 1.6 days compared to 22.2 days for C23-treated (Fig. 6C). At the end of the experiment, LNCaP xenografts treated with C23 were 43% smaller than vehicle-treated ones.

When evaluating plasma PSA levels measured at the beginning and end of the animal study, PSA levels in JHU-LNCaP-SM xenografted mice remained unchanged (terminal concentrations of 3.4 compared to 3.1 ng/mL respectively for control and C23 treated mice; Fig. 6D) despite the clear effect of C23 on JHU-LNCaP-SM tumor growth. This contrasted with results obtained with LNCaP xenografts where PSA levels in terminal plasma samples were 110 and 9 ng/mL respectively for vehicle and C23-treated mice (Fig. 6D). Interestingly, JHU-LNCaP-SM xenografts secreted much lower levels of PSA in the circulation of mice than LNCaP.

Although the JHU-LNCaP-SM did not significantly express GDF-15 when grown in vitro, it was possible to quantitate circulating plasma GDF-15 levels in xenografted mice. Moreover, GDF-15 concentrations were significantly lower in C23-treated mice (975 compared to 443 pg/mL respectively for vehicle and C23-treated mice (Fig. 6E). Correlations between PSA and GDF-15 concentrations with tumor size at the end of the xenograft experiments were examined to determine which circulating marker most closely varied along with PACE4 inhibitory response (Fig. 6F–I). Interestingly, in the JHU-LNCaP-SM xenografted mice, PSA levels did not correlate with the tumor volumes (Fig. 6G, r: 0.50, p = 0.40) whereas GDF-15 positive correlation with tumor sizes was significant (Fig. 6F, r: 0.83, p = 0.0154). In comparison, in LNCaP xenografted mouse plasmas, a very high correlation between circulating PSA levels with tumor size (Fig. 6I, r: 0.94, p = 0.0001), as well as a rather good correlation with GDF-15 were observed (Fig. 6H, r: 0.82, p = 0.0583).

### C23 PACE4 inhibitor treatment induces tumor cell quiescence and apoptosis in JHU-LNCaP-SM xenografts

**Figure 7:**
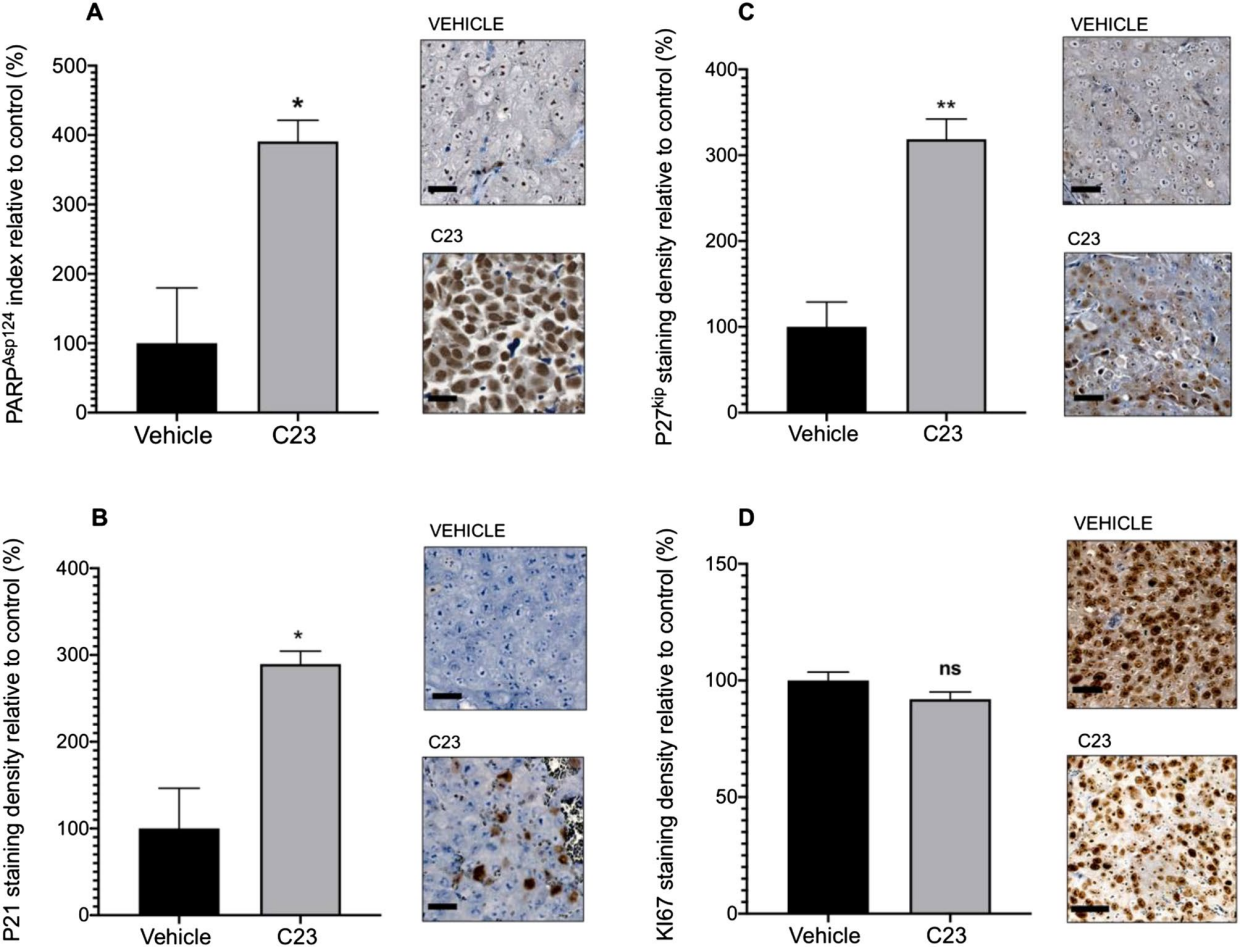
Immunohistological evaluation of xenograft cells apoptosis and quiescence. (**A**) IHC against apoptosis marker cleaved PARP^Asp124^, (**B**) cell quiescence markers p21^WAF^ and (**C**) p27^Kip^, and (**D**) proliferation marker Ki67. Data, means ± SEM (n = 3 using at least 3 fields of > 150 cells each). *p < 0.05; **p < 0.01; *ns* non-significant. Scale bar represents 50 µm.

Immunostainings were performed on FFPE xenografts to identify the rate of tumor cell apoptosis, cell quiescence and proliferation. Using immunohistochemistry against cleaved PARP^Asp124^, an apoptosis marker^17^, a significant increase in the rate of cells undergoing apoptosis in the C23-treated group was observed, with 56% positive cells compared to 15% positive cells in the vehicle group. (Fig. 7A). Using the cell cycle arrest markers p27^KIP^ and p21^WAF^^18^, PACE4-inhibitor treated xenograft showed clearly enhanced quiescence rates with increased index of 28% and 9% p27^KIP^ positive cells C23 and for vehicle respectively (Fig. 7B) and 13% compared to 5% p21^WAF^ positive cells for C23 and for vehicle respectively (Fig. 7C). Still, immunostaining for Ki67, a cell cycle progression marker^19^, did not reveal any significant changes as 67% of cells were positive in control tumors compared to 63% in the C23 group (Fig. 7D).

## Discussion

The androgen axis plays an important role in PCa disease as prostate cells growth depends on testosterone and dihydrotestosterone, which are the hormones targeted by chemical castration therapies^20^. This therapeutic avenue derives from C. Huggins’s finding in 1941 which highlighted the effect of androgen deprivation on PCa. Until today, this has been the basis of most pharmacological intervention prescribed, post-surgery or post-radiation to PCa patients^20^, either by the acting on the endocrine system or directly on androgen synthesis pathways and/or receptor blockade^21^. Although effective, hormone therapies tend to lead to cancer cells adapting to lower levels of circulating androgens and thus lead to androgen independence and further recurrence with often increased aggressivity^2^. While most PCa clinical targets are impacting the androgen axis, the identification of unexplored pathways that are not under the control of androgens may be relevant for the development of novel therapeutic approaches^20,22^. This could be especially important in advanced disease such as castrate resistant PCa that threatens the life of patients.

For novel drug therapies to reach clinical stages for PCa patients, evidence of improved tumor outcomes compared to standards of care is required. In the case of PCa, treatments are mostly tested in late-stage tumors as early-stage tumors are as much as possible handled by prostatectomy which attempts to eliminate the tumor before it spreads. It is thus critical to address whether a novel target such as PACE4 and its pharmacotherapies are likely to be efficient on tumor representative of the condition to treat. PCa pathology can be heterogeneous from one patient to another, and it is therefore difficult to work with a single cell model that mimics the entire spectrum of PCa^4^. The work performed so far demonstrated efficacy using either peptide inhibitor or gene repression in LNCaP and DU145 cell models^7,11,16,23,24^, but these models both have disadvantages for in vivo work. Still, LNCaP had been the central model used to screen compounds and establish key evidence.

Given the interesting properties of the JHU-LNCaP-SM cell model, which is more representative of advanced PCa and more convenient to work with, this model was compared to its parental line; LNCaP. Both cell lines express AR mRNA (Fig. 3C), and according to Castanares et al.^7^, JHU-LNCaP-SM cells express the 120 kDa full-length protein just like LNCaP. Both cell lines contain the characteristic T877A mutation which has been found to be present in clinical samples from advanced PCa patients. However, they do not express the 75 kDa AR-v7 variant of AR^9^. Nevertheless, their sensitivity to androgen significantly differs (Supplementary Fig. 1).The JHU-LNCaP-SM model is thus considered as a late-stage disease model to test the efficacy of novel therapeutic approaches such as PACE4 pharmacotherapy.

To establish the usefulness of these cells in this study, we demonstrated that similarly to LNCaP cells, JHU-LNCaP-SM expresses both PACE4 isoforms (Fig. 1) and are just as responsive as LNCaP in cell-based assays to both the C23 inhibitor and siRNA silencing of PACE4-altCT (Figs. 3, 4). siRNA silencing of PACE4-FL, the consensual isoform of PACE4, did not affect cell growth and colony formation (Fig. 4A–C), thus further reinforcing the concept that PACE4-altCT is the pro-oncogenic driver of PACE4-associated growth supporting the PCa phenotype. When xenografted in nude mice, JHU-LNCaP-SM displayed a take-rate of about 95%, thus far exceeding the one typically observed with LNCaP cells^9^. Moreover, these xenografts secreted PSA as well as GDF-15, which is an important feature of PCa, especially in the context of PACE4 targeting.

So far, the main mechanism of action of PACE4 inhibitors demonstrated in this study is the inhibition of PACE4-altCT isoform as it supports the growth of tumors through notably the processing of secreted autocrine growth factors such as GDF-15^11,25^. This growth factor is part of the TGF-β family of cytokines and its high expression is often associated with cancer progression. It is suggested that tracking the degree of PACE4 inhibition by this biomarker represents an relevant avenue for prostate cancer therapy efficacy^26^.

Interestingly, despite low levels of GDF-15 secreted by the JHU-LNCaP-SM cells in vitro (Fig. 5B), when xenografted they clearly secrete significant amount of circulating GDF-15 which even correlate better with tumor size than PSA (Fig. 6E,F). In LNCaP xenografts, the observed good correlation between PSA and tumor size as already been documented and is used in several studies^27^. This discrepancy is likely the reflection of the androgen independence of JHU-LNCaP-SM cells, which secretes little PSA compared to LNCaP (Fig. 6D). We hypothesize that enhanced GDF-15 correlation with tumor response in this study is due to the direct relationship between the drug target and its substrate processing, making secreted GDF-15 a PACE4 target-engagement biomarker.

## Conclusion

The JHU-LNCaP-SM model enhances our understanding of the wide spectrum of PCa disease progression. This particular model may prove useful in the pharmacokinetic and pharmacodynamic characterizations of PACE4 targeting inhibitors. This is justified by the fact that JHU-LNCaP-SM cells express all the prerequisites needed like PACE4-altCT isoform, pro-GDF15 as a specific PACE4 substrate, and sensitivity for C23 peptide comparable to the parental LNCaP line. While we have not explored other potential therapeutic pathways, likely, JHU-LNCaP-SM will also be an important tool for these types of experimentation.

## Supplementary Information

Supplementary Information.

## Data Availability

Raw data generated or analyzed during this study are included in the Supplementary Information or available from the corresponding author upon request.
