# Supplementary material for: Efficacy of PACE4 pharmacotherapy in JHU-LNCaP-SM preclinical model of androgen independent prostate cancer
Source: Sci Rep. 2022 Oct 19;12:17489. doi: 10.1038/s41598-022-21593-7 (PMC9582213; doi:10.1038/s41598-022-21593-7)
Supplement: Supplementary file 1 — Supplementary Information. [file 41598_2022_21593_MOESM1_ESM.pdf]

*Supplementary information*

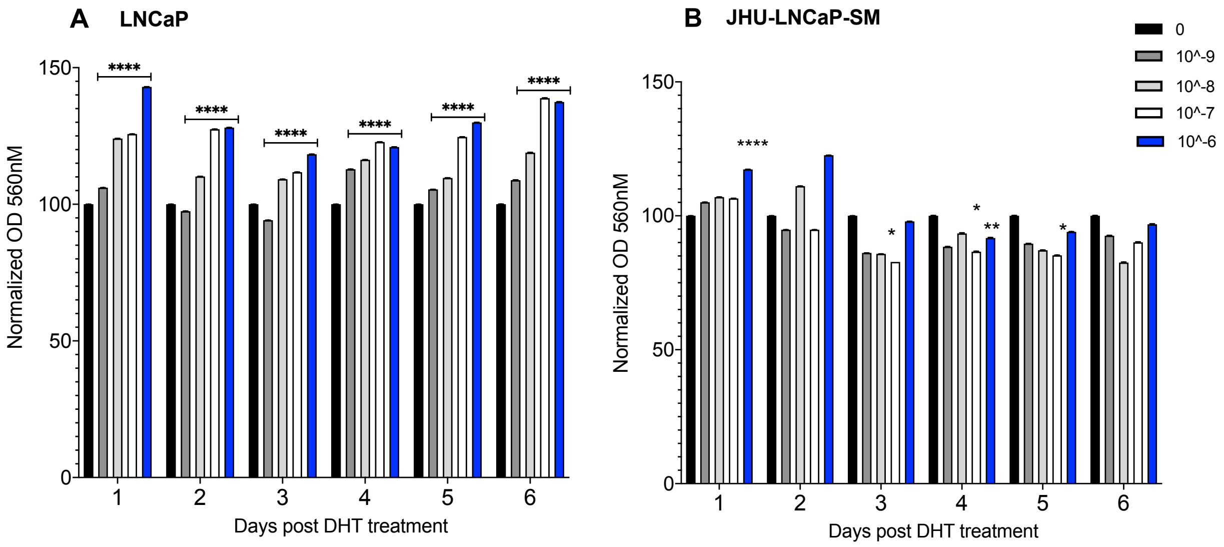


***Supplementary Figure 1:*** **JHU-LNCaP-SM cells are unaffected by the levels of dihydrotestosterone. (A)** MTT assay in LNCaP and (**B**) JHU-LNCaP-SM cells supplemented with different DHT concentration (0, $10^{-9}$ ,$10^{-8}$,$10^{-7}$,$10^{-6}$ M) over 6 days. Data are means ± SEM (n=4). **p<0,01 from a Student t-test.

Table 1 – Primers used for RT-qPCR reaction

| Genes | Protein | Sequence |
|---|---|---|
| PCSK6 | PACE4-FL | Fw : 5’-ACCCAGAAGAGATGCCGG-3’<br>Rv : 5’-ACCATCTCGCAGAATGTCTCG-3’ |
| PCSK6 | PACE4-altCT | Fw : 5’-ACCCAGAAGAGATGCCGG-3’<br>Rv : 5’-TTCTCAACCTCGGCACTATTG-3’ |
| *PCSK3* | furin | Fw : 5’-GTGGCGACCTGGCCATCCAC-3’<br>Rv : 5’-AGGTACGGGCAGCCCCTCAG-3’ |
| *PCSK1* | PC1/3 | Fw : 5’-GCCCACACAAGAGAACCCTAAG-3’<br>Rv : 5’-CTCCCTCCTCCAACTCATCCC-3’ |
| *PCSK2* | PC2 | Fw : 5’-AAAGAAGGATGAAGGGTGGTTGTG-3’<br>Rv : 5’-GCTGCTGCTTGTGGTGTAGG-3’ |
| *PCSK5* | PC5/6 | Fw : 5’-GCAGCCATATTCACCAACCAATG-3’<br>Rv : 5’- CCGTCACAGCCAACCTCAC-3’ |
| AR | AR | Fw : 5’GACATGCGTTTGGAGACTGC-3’<br>Rv : 5’- GTTGTTGTCGTGTCCAGCAC-3’ |
| GDF15 | GDF-15 | Fw : 5’-AACTCAGGACGGTGAATGGC -3’<br>Rv : 5’- AGCCGCACTTCTGGCG -3’ |

Table 2 - Antibodies used and their specific conditions

| Target | Species | Manufacturer | Conditions | Clone or # |
|---|---|---|---|---|
| Ki67 | Rabbit | Cell Signaling Technologies | IHC: 1/400 | D2H10 |
| Cleaved $PARP^{Asp214}$ | Rabbit | Cell Signaling Technologies | IHC: 1/50 | D64E10 |
| $p27^{KIP}$ | Mouse | DAKO | IHC: 1/50 | SX53G8 |
| P21 | Mouse | Cell Signaling Technologies | IHC: 1/50 | DCS60 |

Uncropped raw blots from Figure 5C

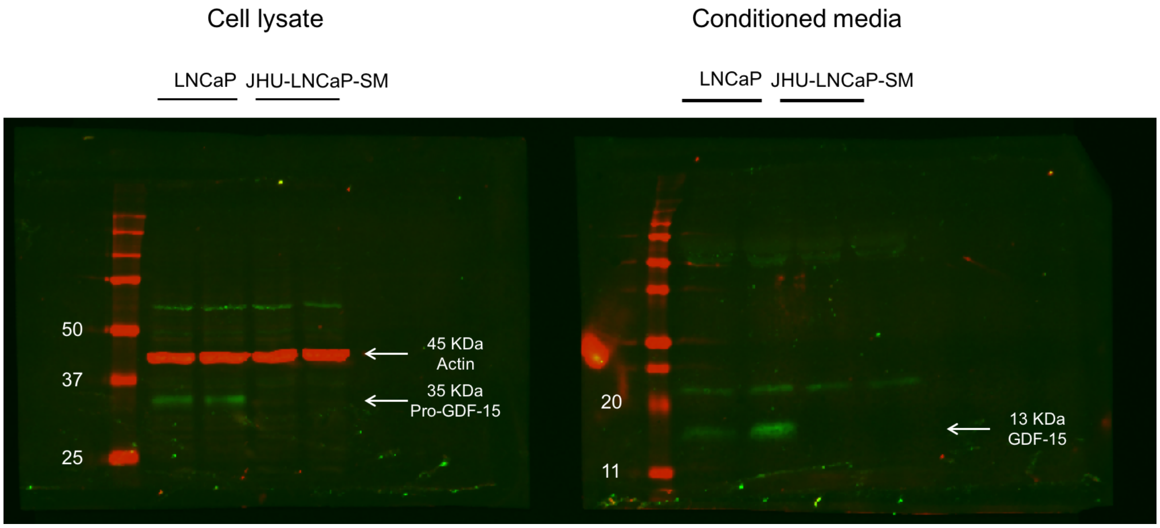


Xenograft assay

All studies were performed in accordance with the Canadian Council on Animal Care guidelines and were approved by the University of Sherbrooke animal Ethical Care Committee. Protocols numbers: 2016-1997 and 2020-2701.

Animals euthanasia

Animals are anesthetized by ketamine:xylazine (0.2 mL/100 g ip dosing of 87:13 mg/kg respectively) injection and cardiac exsanguination is realized. After, a cervical disruption was performed to ensure the mouse sacrifice.
